# Atypical Rasmussen's Encephalitis

**DOI:** 10.7759/cureus.46647

**Published:** 2023-10-07

**Authors:** Maria A Alfonso, Martha C Piñeros-Fernández, Luisa F Jaimes, Nicolas I Ramos

**Affiliations:** 1 Pediatrics, Los Cobos Medical Center, Bogotá, COL; 2 Pediatric Neurology, Los Cobos Medical Center, Bogotá, COL; 3 Pediatric Neurology, Fundación Cardioinfantil - La Cardio, Bogotá, COL; 4 Neuroradiology, Los Cobos Medical Center, Bogotá, COL

**Keywords:** brain magnetic resonance images, language regression, seizure, refractory epilepsy, focal motor deficit, hemiplegia, autoimmune encephalitis, encephalitis, case report, rasmussen syndrome

## Abstract

A three-year-old female patient was admitted to our institution due to subacute fever, intermittent vomiting, persistent bilateral mydriasis after cycloplegia, right central facial palsy, and mild right hemiparesis with hyperreflexia. Brain MRI shows encephalitis in frontal, parietal, insular, and left putamen course and loss of cortical volume and white matter of the entire left hemisphere which are features described in Rasmussen's encephalitis (RE). Therapy with intravenous methylprednisolone bolus was initiated, with adequate clinical response. We consider in this case the diagnosis of atypical RE by imaging criteria in the subacute stage. There are few reports of atypical RE without epilepsy or continuous partial epilepsy. Our purpose is to present a case of a patient with RE images without epilepsy seizures and review the diagnostic and therapeutic approach of RE.

## Introduction

Rasmussen's encephalitis (RE) is a rare progressive neurologic disorder characterized by unilateral atrophy of the cerebral cortex, refractory focal seizures, progressive hemiparesis, and cognitive deficit. Neuropathological studies have demonstrated the theory of a T-cell response to one or more antigenic epitopes that mediate the onset of brain damage, which may be a window of treatment for future therapies [1]. RE is a chronic and progressive disorder, with refractory focal seizures and even continuous partial epilepsy, progressive unilateral motor function impairment, and cognitive compromise. The estimated annual incidence is 1.7 cases per 10 million people under 16 years of age, it is more frequent in children between 6 and 8 years of age, but it can also affect adolescents and adults. Any relation with sex, geographical area, or ethnic group has not been identified [1,2].

RE is defined as an immune-mediated disease, with the constant involvement of T cells and histopathological findings of inflammation in cortical multifocal areas, associated with perineuronal lymphocytic infiltration, T cells (which are fundamental in brain damage), and microglial activation [3]. Subsequently, CD8+ T cytotoxic cells generate the activation of the inflammation pathway [4]. Therefore, the release of IL-1, the release of granzymes, responsible for neuronal apoptosis [4], CD4+ T-cell clones, γδ T cells secreting IFN-γ and TNF, and cytokine activation are also involved [5,6]. The diagnosis of RE is based on clinical, electrophysiological, and morphological characteristics [1].

The European consensus developed in 2005 by a panel of experts proposed the RE diagnostic criteria which are still accepted and used today. The criteria in Part A are typical of the early cases including i. clinical focal epilepsy with or without continuous partial epilepsy (CPE) and unilateral cortical deficit, ii. EEG with unihemispheric slowing with or without epileptiform activity and onset of unilateral seizures, and iii. MRI with unihemispheric focal cortical atrophy and at least one of the following: hyperintensity signal of gray or white matter T2/FLAIR w/o hyperintensity signal or atrophy of caudate head ipsilateral [1,2]. As per criteria, any one of A criteria or all three is needed, if the patient does not fulfill part A criteria, two of the three criteria of part B should be met, which are likely to cover early cases and less common presentations, including i. Clinical characteristics of CPE or progressive unilateral cortical deficit, ii. Progressive unihemispheric focal cortical atrophy, and iii. Histopathological features of T-cell-dominated encephalitis with activated microglial cells typically, but not necessarily forming nodules and relative astrogliosis; numerous parenchymal macrophages, B cells or plasma cells, or viral inclusion bodies exclude the diagnosis of RE [1,2].

## Case presentation

We present the case of a previously healthy three-year-old female patient; she was born at 40 weeks from the first normal gestation of healthy parents by cesarean section indicated by the cord wrapped around the baby's neck leading to unsatisfactory fetal status. Spontaneous neonatal adaptation, adequate weight, and height at birth were present. There was a family history of second-degree relatives with epilepsy. Normal acquisition of motor and language developmental skills.

**Figure 1:**
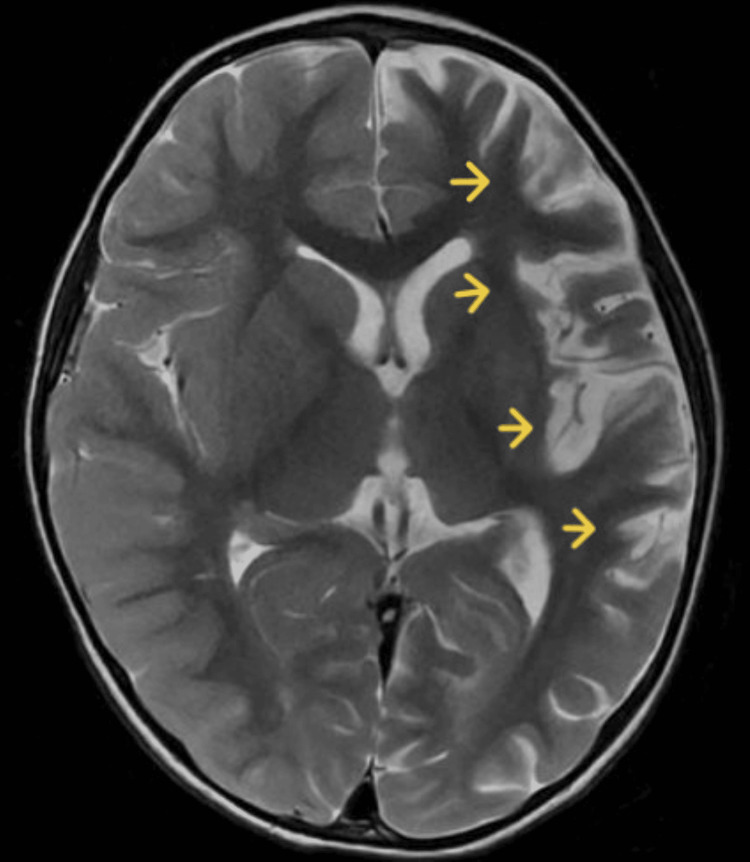
First brain MRI axial T2

**Figure 2:**
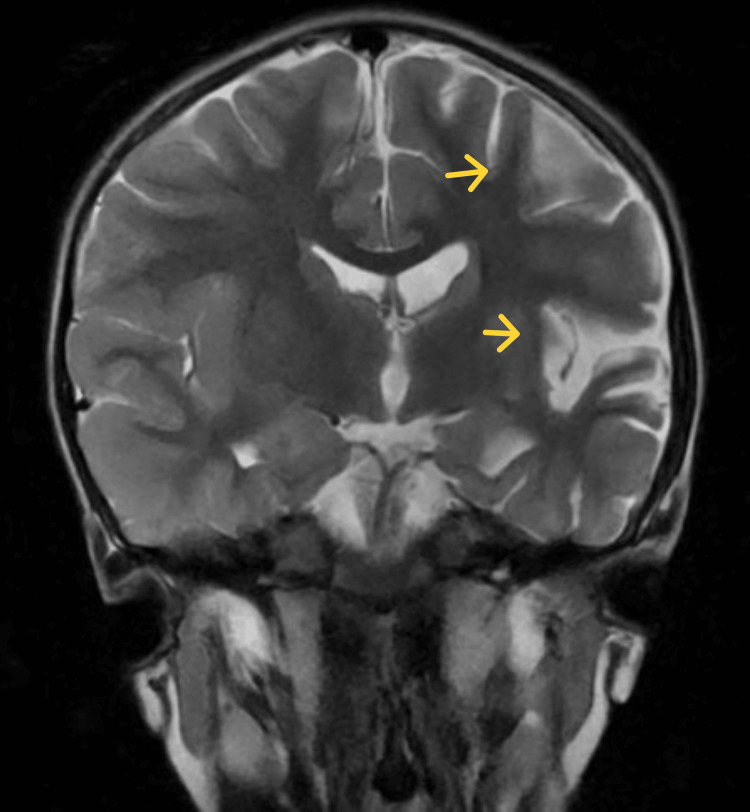
First brain MRI coronal

The patient was brought to the ophthalmologist consultation for intermittent strabismus and fundus examination which described normal but for persistent mydriasis after application of cycloplegic eye drops; the patient was transferred to the ambulatory Pediatric Neurology service. At the consultation with the pediatric neurologist, the parents also reported that the patient presented repeated episodes of vomiting, asthenia, and abnormal speech. On the clinical examination, convergent strabismus of the left eye with bilaterally non-reactive mydriatic 4 mm pupils was reported and the first brain MRI was ordered (Figures 1, 2) which was reported with increased depth of cortical sulci of the left cerebral hemisphere on frontal, parietal, temporal and insular with gliosis and atrophy of the cortex, without defining signs of ischemic involvement in vascular territory or acute inflammatory, and the patient was referred to our institution.

At the admission to our institution, expanding the information, it was known that the patient presented vomiting associated with fever which had been going on for a total of two months, continuous fever of 38.5°C, left deviation of angle of mouth to left, asthenia, hyporexia, and vomiting n° three a day during the three days before the admission. On the clinical examination, the patient was febrile and alert, with no meningeal signs, normal speech, bilateral cicloplegic mydriasis, strabismus, right facial palsy, and mild right-side hemiparesis and right-side hyperreflexia.

**Figure 3:**
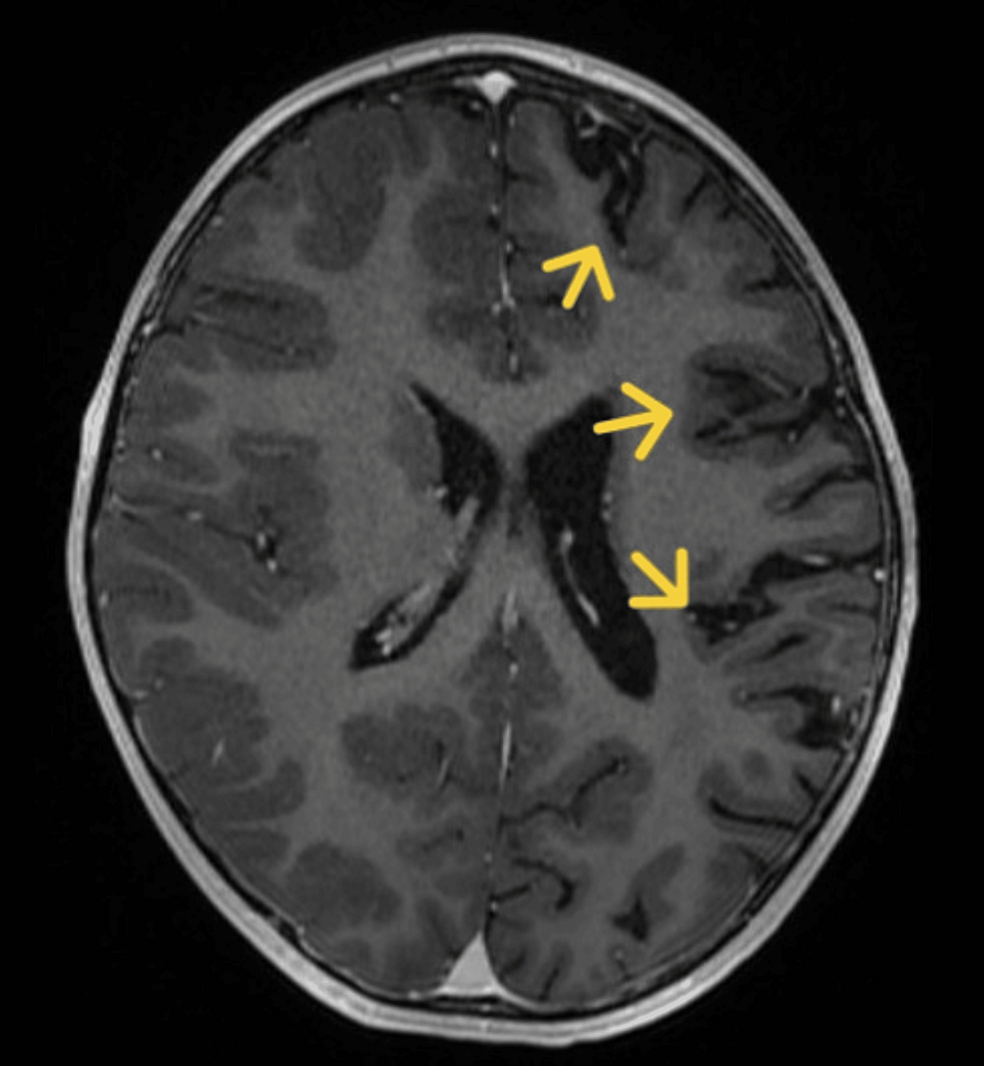
Second brain MRI with contrast Decrease of the left hemisphere volume. Note that the frontal, parietal, and temporal lobes are the most affected as observed in the first MRI.

**Figure 4:**
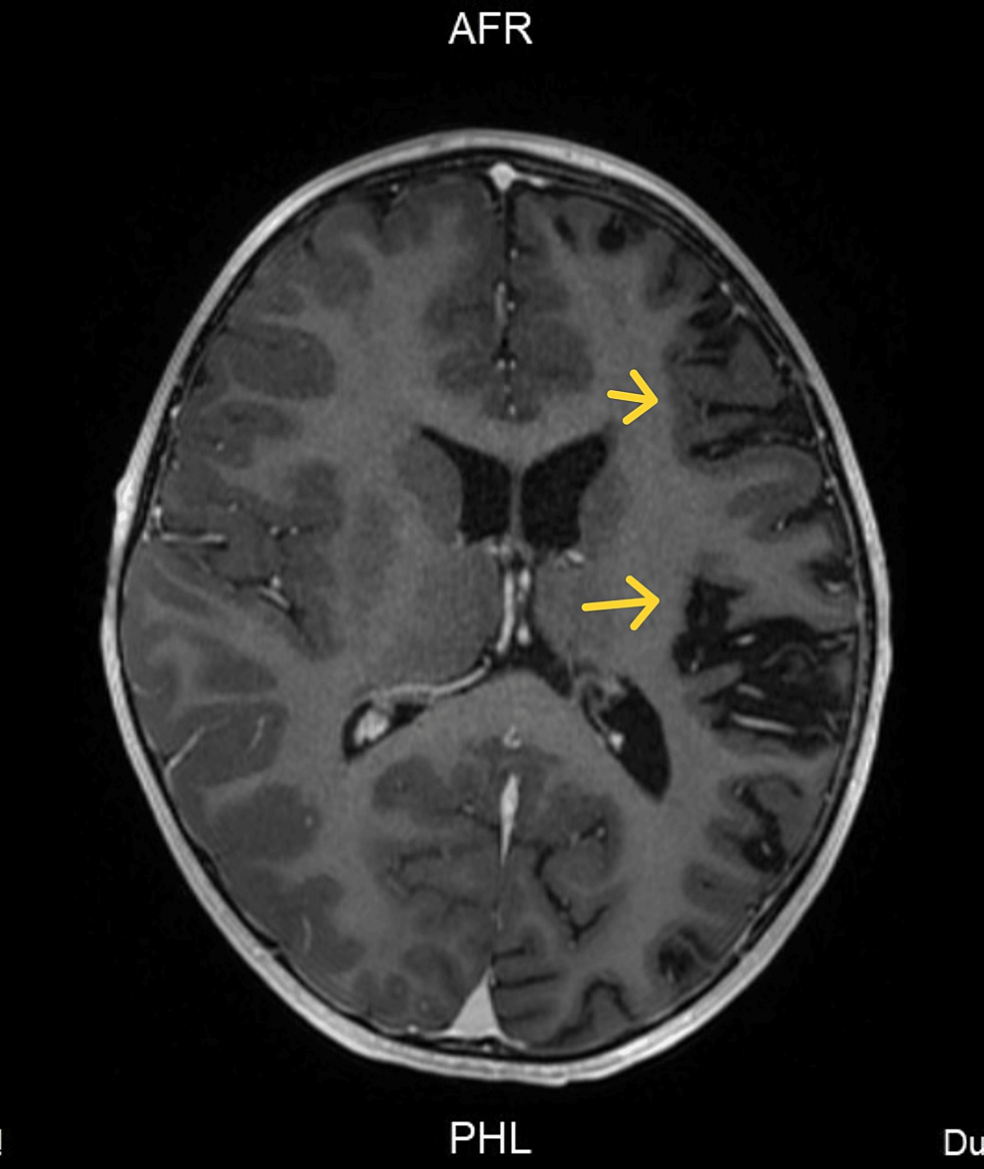
Second brain MRI with contrast axial section Non-enhancement observed on the left hemisphere.

**Figure 5:**
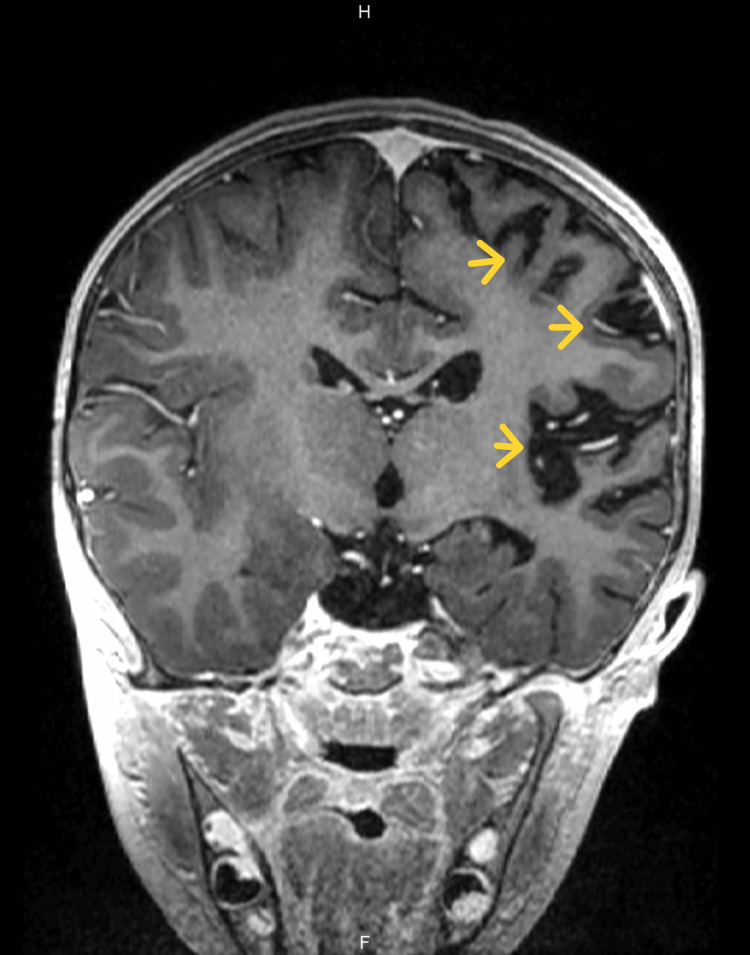
Second brain MRI, contrasted study, coronal section This figure details marked compromise of the insular region of left hemisphere also observed in the first brain MRI of the patient.

**Figure 6:**
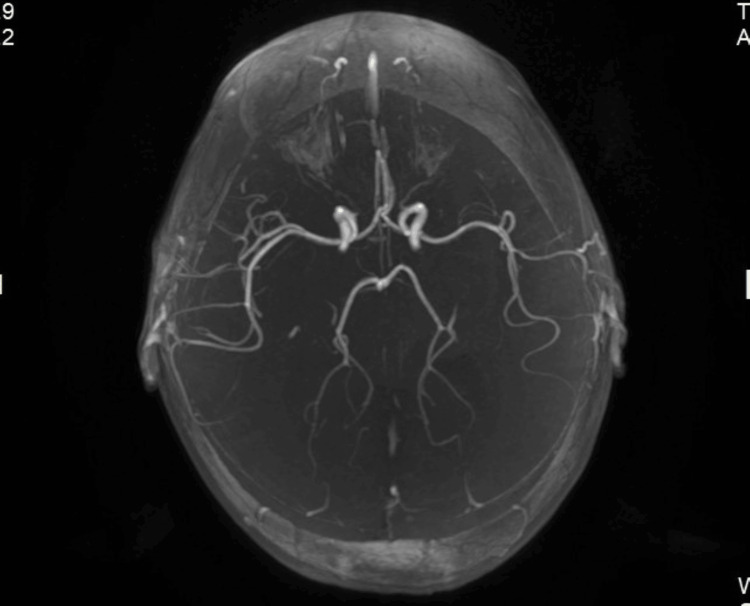
Normal brain arterial angioresonance

**Figure 7:**
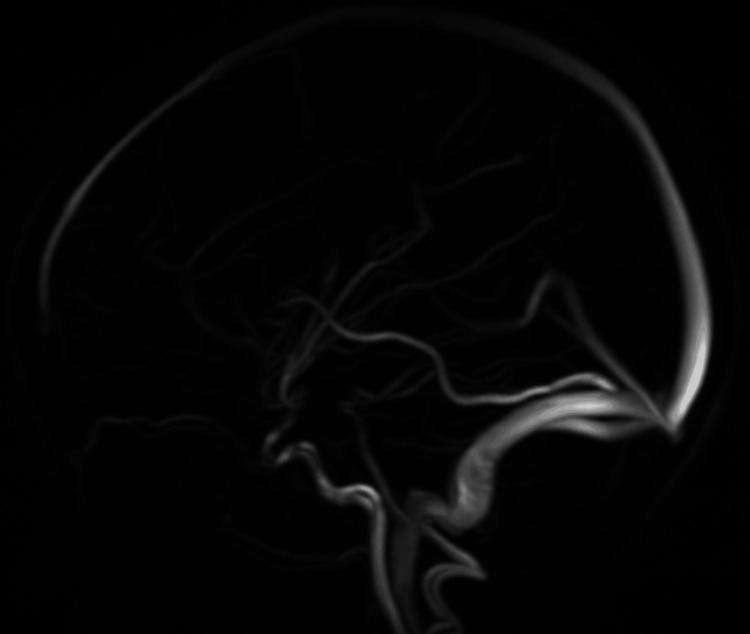
Normal brain venous angioresonance

**Figure 8:**
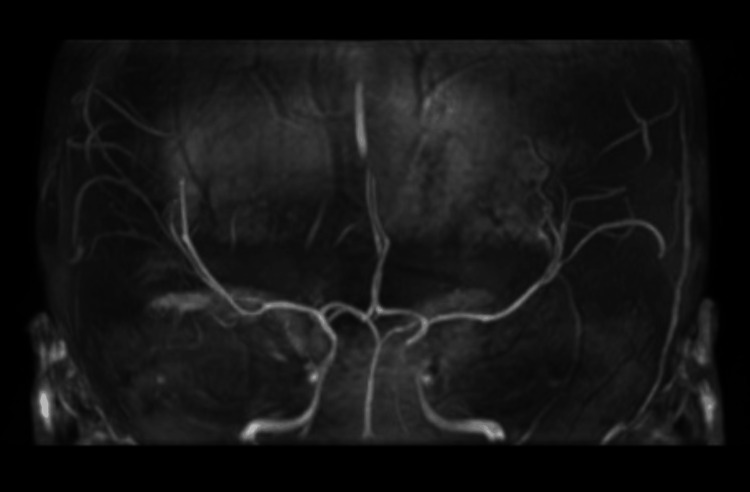
Normal brain arterial angioresonance coronal section

**Figure 9:**
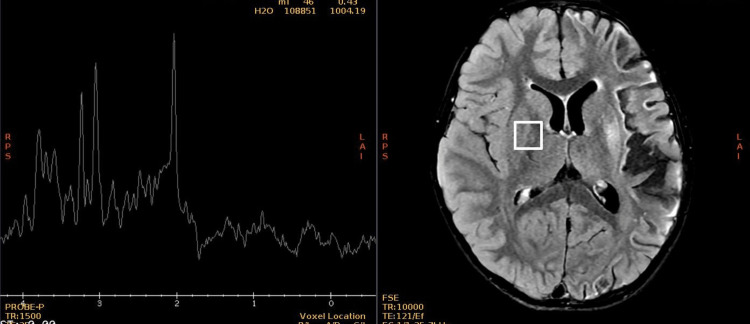
Normal brain MR spectroscopy of the right hemisphere

**Figure 10:**
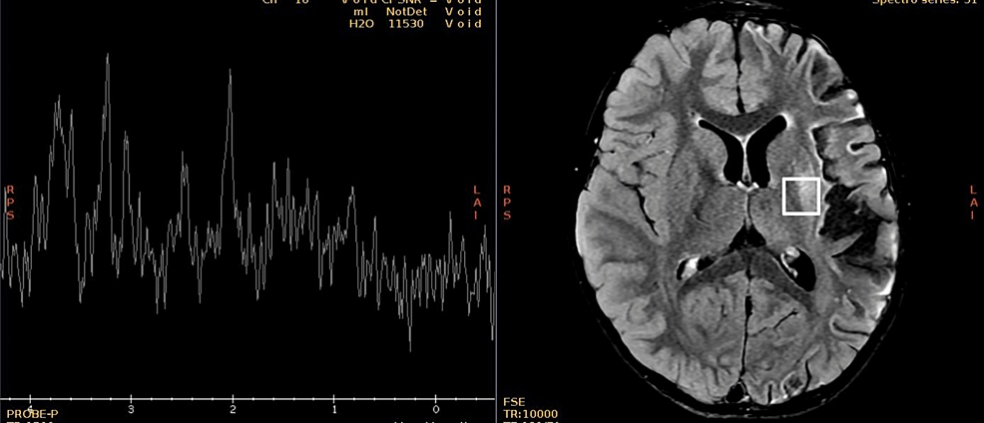
Abnormal brain MR spectroscopy of the left hemisphere Complementary study with brain spectroscopy for hydrogen ions H+, of brain metabolites in short and intermediate echo time (ET) in the region of interest that shows diffuse decrease of all metabolites, without defining the presence of lactate or lipids, which in comparison with the contralateral area, diffuse decrement in neuronal population, and reduction of choline and creatine peaks, which correlates with gliosis changes.

A new contrasted brain MRI and orbit MRI studies (Figures 3-5) were indicated, and the report was an asymmetric decrease of the cortical volume of the left cerebral hemisphere. Angioresonance with arterial and venous phases was normal (Figures 6-8) and the brain spectroscopy (Figures 9, 10) showed a reduction of choline and creatine peaks consistent with gliosis. MRI of the orbits for optic nerve study was normal. 

**Figure 11:**
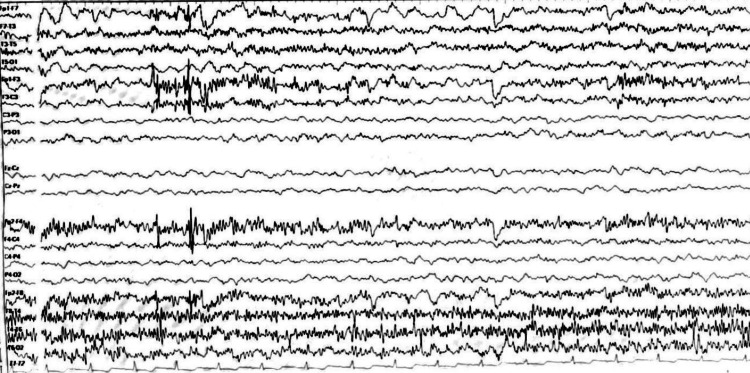
EEG The  electroencephalographic record was normal

Figures 6, 7 show a normal patient`s angioresonance. Without defined alteration in the caliber of the arterial or venous vascular structures, no occlusion of the left middle cerebral artery or the left intracranial internal carotid artery was observed. Figures 9, 10 show brain spectroscopy images of the patient. A 12-hour videoelectroencephalograpy (Figure 11) was done, and no seizures and no epileptic abnormalities were observed. 

The results of laboratory studies are presented in Table 1. The patient underwent lumbar puncture obtaining clear and colorless cerebrospinal fluid (CSF), normal CSF cytochemistry, ADA, normal serum and CSF lactate values; and negative film array and cultures for pathogens in CSF discarding concomitant infection of the central nervous system.

**Table 1:** Laboratory results CSF: Cerebrospinal fluid; ADA: adenosine deaminase activity

Variable	Results	Normal
Blood leukocytes	11.070 x 10^3/uL	5.500 – 15-500
Blood neutrophils	8.060 x 10^3/uL	1.600 – 8.290
Blood lymphocytes	2.060 x 10^3/uL	3.000 – 9.000
Blood platelets	250.000 x 10^3/uL	150.000 – 450.000
Hemoglobin	12.100 g/dl	9.5 – 15.5
Hematocrit	35.4 %	33 – 41 %
TGO	30.0 U/L	5 – 32 U/L
TGP	11.0 U/L	5 – 33 U/L
C3	104.4 mg/dL	90 -180 mg/dL
C4	29.6 mg/dL	10 - 40 mg/dL
Blood glucose	98 mg/dL	60 - 100 mg/dL
CSF leukocytes	0	0 - 8/mm3
CSF glucose	61 mg/dL	40 - 80 mg/dL
CSF proteins	14.70 mg/dL	10 - 45 mg/dL
CSF pathogens film array	Negative	-
Oligoclonal bands CSF	Negative	-
Serum lactate	1.6 mmol/L	1.1 - 2.4
CSF lactate	Negative	<2 mmol/L
CSF cultures for gram (+) and gram (-)	Negative	-
CSF ADA	0.39 U/L	0 – 9 U/L
Herpes Ig M	0.0	Negative < 0.90 Positive > 1.10
Herpes Ig G	0.2	Negative < 0.90 Positive > 1.10
Anti-Ro	4.9 CU	0 - 20
Anti-La	3.3 CU	0 - 20
Anti-SM	7.9 CU	0 - 20
Anti-RNP	3.5 CU	0 - 20

With the diagnosis of atypical presentation of RE as the first possibility, the treatment initiated was corticosteroids with intravenous bolus of intravenous methylprednisolone, without adverse effects. The patient had no seizures or abnormal movements during the hospitalization. The patient evolved satisfactorily, the fever stopped, the patient remained stable without epileptic seizure symptoms and major functional or cognitive impairment, and the persistent cycloplegic mydriasis observed at the admission was resolved and interpreted as a prolonged pharmacologic effect. At the discharge, oral corticosteroids were indicated at the discharge and multidisciplinary and brain MRI follow-up. 

## Discussion

The present clinical case corresponds to an atypical presentation of RE since no clinical signs suggestive of epileptic seizures have been documented until the patient discharge, correlated with the normal 12 hours videoelectroencephalogram traced of the patient who did not present seizure or ictal event along the register; although epileptiform abnormalities on EEG are common in RE and often develop into electrographic seizures, continuous partial epilepsy is not always accompanied by recognizable ictal surface activity [5]. Particularly, our patient presented clinical cortical deficit given by mild right hemiparesis and central right facial palsy, and general symptoms such as asthenia and fever also have been described in patients with RE [7]. However, the main feature in the MRI could be explained by RE with an asymmetric decrease in the volume of the cortex of the left cerebral hemisphere, with increased involvement of the frontal, parietal, temporal, and insular lobes and gliosis in the early stage given by hypersignal in the FLAIR sequence in the insula and left putamen.

Brain MRI arises as a very useful diagnostic and follow-up imagenologic study in RE. It has been described in the acute stage that unilateral enlargement of the ventricular system in T2 and FLAIR hyperintensity is observed at the cortical or subcortical level, the perisylvian region being the site of predilection for signal change and volume loss; additionally, ipsilateral cortical atrophy of the head of the caudate nucleus accompanying hemispheric atrophy is usually an early sign [6,8]. Other studies should be performed in RE, as studies on CSF and blood exclude infections of the central nervous system or other associated disorders. To our knowledge, there are no specific biochemical biomarkers for RE, but there are studies that have detected different viral particles among which is the herpes virus; however, Herpes IgG and IgM antibodies were negative, and CSF analysis was normal in the present case.

The clinical course of RE presents three stages: the first stage is the "prodromal stage", an average duration of 7.1 months, characterized by nonspecific and infrequent seizures associated with mild hemiparesis. In subsequent months, all patients experience the second stage the "acute stage" which is based on focal motor seizures or CPE characterized by being an intractable focal somatomotor status epilepticus, continuous focal spasms of a part of the body (distal limb or face) and with cortical origin. This stage has an average duration of 25 months. The last stage is the "residual stage" characterized by less frequent epileptic seizures and persistent and stable neurological deficits [1].

Furthermore, it is necessary to closely follow our patient to detect clinical manifestations such as late seizures or abnormal movements, but we expect that the inflammatory immune-mediate process of RE becomes controlled. However, ER patients without or with delayed seizure onset have been published. There are four reports of atypical RE patients without seizures or delayed seizure onset [9-12]. In a retrospective study of 10 patients with atypical RE, the most frequent feature was progressive hemiparesis without seizure or late-onset events, followed by dyskinetic movements, late-onset seizures associated with unilateral caudate atrophy, image mimicking focal cortical dysplasia as the first MRI abnormality, and cluster epileptic spasms, only one patient remained without seizures [11]. There are two reports of RE patients who did not present seizures (Table 2) [13,14]. In one report, the patient had dual epileptogenic pathologic conditions, cortical dysplasia, and RE [13], and in the other report, the patient presented relapsing-remitting bilateral RE without seizures [14]. 

**Table 2:** Reports of atypical pediatric presentations of RE with delayed seizures or without seizures ^a^ Dual pathology (cortical dysplasia); ^b^ Bilateral relapsing-remitting RE RE: Rasmussen's encephalitis

Author	Year	N	Age (years)	Nº delayed seizure	Nº without seizures	Ref
Bien	2007	5	1.3 - 1.9	2	3	[9]
Ferrari	2011	1	5.5	1	-	[10]
Ravindra^ a^	2015	1	12	-	1	[13]
Caraballo	2018	10	2 - 15	9	1	[11]
Noordin	2021	1	17	1	-	[12]
Liu^b^	2023	1	9	-	1	[14]

Currently, anticonvulsant medications, immunotherapy, and surgery are the three fundamental pillars in the treatment of RE. Based on the neuroinflammatory pathological basis of RE, immunomodulatory therapy including a high dose of corticosteroids as the first-line treatment, intravenous immunoglobulins, plasmapheresis, rituximab, and oral immunosuppressor medications such as azathioprine and tacrolimus are used [2,15,16]. Early immunomodulatory therapy has benefits such as inhibiting seizure episodes, preserving neurological function, and delaying the requirement for surgery; however, it can potentially be associated with loss of functions represented by the affected hemisphere. However, anticonvulsant medications alone or in combination have a limited effect on RE [17]. Hemispherectomy is considered as a definite surgical treatment for intractable focal epilepsy in RE, but the timing of surgery for a better cognitive outcome is controversial. Disconnective hemispherectomy reduces the risk of hydrocephalus and shunting compared with anatomic hemispherectomy [18].

## Conclusions

Atypical RE without seizures should be considered in pediatric patients with abnormalities in MRI not explained by other brain disorders. The clinical presentation of cortical deficit can be subtle as the present case with mild hemiparesis and facial central palsy. Importantly, other relevant issues were discarded on the facial examination of our patient such as cutaneous lesions, angiomas, subcutaneous or dermis atrophy, and abnormalities or deformities of craniofacial bones. We reassured that the facial aspect and symmetry in the recent and previous photographs of the patient were normal. Therefore, the early setting of immunomodulatory treatment mitigates the progressive deterioration of cognitive and motor functions and intractable epilepsy, the most frequent manifestations in the pediatric population with RE, leading to disability at an early age with important social impact. Nevertheless, the literature has shown the use of immunomodulatory therapy, which slows the progression of the disease but does not exclude the possibility of requiring hemispherectomy.
